# Integrative Analysis Between Genome-Wide Association Study and Expression Quantitative Trait Loci Reveals Bovine Muscle Gene Expression Regulatory Polymorphisms Associated With Intramuscular Fat and Backfat Thickness

**DOI:** 10.3389/fgene.2022.935238

**Published:** 2022-08-04

**Authors:** Bárbara Silva-Vignato, Aline Silva Mello Cesar, Juliana Afonso, Gabriel Costa Monteiro Moreira, Mirele Daiana Poleti, Juliana Petrini, Ingrid Soares Garcia, Luan Gaspar Clemente, Gerson Barreto Mourão, Luciana Correia de Almeida Regitano, Luiz Lehmann Coutinho

**Affiliations:** ^1^ Department of Animal Science, College of Agriculture “Luiz de Queiroz”, University of São Paulo, Piracicaba, Brazil; ^2^ Department of Agroindustry, Food, and Nutrition, College of Agriculture “Luiz de Queiroz”, University of São Paulo, Piracicaba, Brazil; ^3^ Embrapa Pecuária Sudeste, São Carlos, Brazil; ^4^ College of Animal Science and Food Engineering, University of São Paulo, Pirassununga, Brazil

**Keywords:** backfat thickness, carcass and meat quality, expression quantitative trait loci, intramuscular fat content, RNA-Seq, SNP, Nellore cattle

## Abstract

Understanding the architecture of gene expression is fundamental to unravel the molecular mechanisms regulating complex traits in bovine, such as intramuscular fat content (IMF) and backfat thickness (BFT). These traits are economically important for the beef industry since they affect carcass and meat quality. Our main goal was to identify gene expression regulatory polymorphisms within genomic regions (QTL) associated with IMF and BFT in Nellore cattle. For that, we used RNA-Seq data from 193 Nellore steers to perform SNP calling analysis. Then, we combined the RNA-Seq SNP and a high-density SNP panel to obtain a new dataset for further genome-wide association analysis (GWAS), totaling 534,928 SNPs. GWAS was performed using the Bayes B model. Twenty-one relevant QTL were associated with our target traits. The expression quantitative trait loci (eQTL) analysis was performed using Matrix eQTL with the complete SNP dataset and 12,991 genes, revealing a total of 71,033 cis and 36,497 trans-eQTL (FDR < 0.05). Intersecting with QTL for IMF, we found 231 eQTL regulating the expression levels of 117 genes. Within those eQTL, three predicted deleterious SNPs were identified. We also identified 109 eQTL associated with BFT and affecting the expression of 54 genes. This study revealed genomic regions and regulatory SNPs associated with fat deposition in Nellore cattle. We highlight the transcription factors *FOXP4, FOXO3, ZSCAN2,* and *EBF4,* involved in lipid metabolism-related pathways. These results helped us to improve our knowledge about the genetic architecture behind important traits in cattle.

## Introduction

Over the last years, several studies have shown a growing interest in understanding the molecular mechanisms regulating carcass and meat quality traits in beef cattle (Cesar et al., 2014; Fernandes Júnior et al., 2016; Silva-Vignato et al., 2017; Park et al., 2018; Raza et al., 2020). Carcass and meat quality fat traits, such as backfat thickness (BFT) and intramuscular fat content (IMF) are economically important for the beef industry since they affect the yield of cuts, dressing percentage, and the final consumer perception of meat quality (Yokoo et al., 2008; Park et al., 2018). BFT and IMF are traits related to the final amount of fat in the carcass and play an important role in the determination of meat palatability. The subcutaneous fat layer protects the carcass in the cooling process, minimizing evaporative weight loss and avoiding muscle fiber cold-shortening (Yokoo et al., 2008). The IMF content, also known as marbling, affects beef juiciness, tenderness, and palatability, relevant sensory characteristics for the consumers (Troy et al., 2016; Park et al., 2018). Moreover, the IMF is composed of higher levels of polyunsaturated fatty acid (PUFA) and monounsaturated fatty acid (MUFA), which are beneficial for human health (Troy et al., 2016).

Fat deposition in beef cattle depends on several intrinsic and extrinsic factors, such as the stage of growth, physiological maturity, nutrition, and genetics (Kauffman and Berg, 2011). Researchers have already explored some of these factors showing the differences in body composition among and within breeds (Burrow et al., 2001; Yokoo et al., 2008; Lopes et al., 2012). In the genetics field, genome-wide association studies (GWAS) have been used to detect DNA variants and genomic regions (quantitative trait loci, QTL) associated with carcass and meat quality traits (Tizioto et al., 2013; Cesar et al., 2014; Fernandes Júnior et al., 2016; Raza et al., 2020). Fernandes Júnior et al. (2016), identified genomic regions and putative candidate genes associated with ribeye area and BFT in Nellore cattle. Recently, Martins et al. (2021) found genomic regions on chromosomes 1, 2, 5, 6, 7, 8, 10, 13, 14, and 26, which together explained 12.96% of the total additive genetic variance of fatness (backfat and rump fat thickness) in Nellore cattle. The authors reported seven candidate genes involved in metabolic pathways related to fatness and lipid metabolism (Martins et al., 2021). In previous studies from our research group, Tizioto et al. (2013) found a small effect QTL associated with meat and carcass quality traits in Nellore cattle. Cesar et al. (2014) identified 23 moderate effect QTL associated with fatty acids composition and small effect QTL associated with intramuscular fat in Nellore cattle. Although GWAS reveal genomic regions and putative candidate genes associated with the phenotypes, such analysis provides limited information on the molecular regulation of phenotypes (Michaelson et al., 2009).

Understanding the architecture of gene expression is fundamental to unraveling the molecular mechanisms regulating complex traits (Spielman et al., 2007; Lee, 2018). In previous studies from our lab, we have identified differentially expressed genes in the muscle transcriptome of Nellore cattle with extreme values for BFT and IMF, revealing metabolic pathways and biological processes involved with these traits (Cesar et al., 2015; Silva-Vignato et al., 2017). We also detected modules of co-expressed genes correlated with BFT in Nellore cattle, underling relevant pathways involved in bovine fat deposition (Silva-Vignato et al., 2019). These studies helped us to gain insights into how gene expression influenced these phenotypes. However, there are still gaps in our knowledge about gene expression regulation in cattle.

The expression quantitative trait loci (eQTL) mapping effectively integrates genetic variations and gene expression at the whole-genome level (Westra and Franke, 2014). eQTL data provide substantial insights into transcriptional regulation, functional interpretation for trait-associated SNP, and genetic factors that regulate a specific disease or a complex phenotype (Michaelson et al., 2009; Shabalin, 2012; Westra and Franke, 2014). Thus, our main goal was to identify gene expression regulatory polymorphisms within genomic regions associated with intramuscular fat and backfat thickness in Nellore cattle. To achieve this goal, GWAS and eQTL analyses were performed using an SNP dataset formed by transcribed variants mined from RNA-Seq data combined with a high-density panel of SNP.

## Materials and Methods

### Animals, Samples, and Phenotypes

The experimental procedures related to animal handling and care were approved by the Institutional Animal Care and Use Committee Guidelines from EMBRAPA (CEUA 01/2013).

A population of 193 Nellore steers, derived from an experimental herd of the Brazilian Agricultural Research Corporation (EMBRAPA), and originated from 34 unrelated bulls representing the principal Brazilian Nellore genealogies, was used in the current study. Between the years 2009 and 2011, the animals were raised in grazing systems and finished in feedlots with the same handling and nutritional conditions. The steers were slaughtered at an average age of 25 months and 452 kg in a commercial slaughterhouse located in Bariri (São Paulo, Brazil), following the Brazilian Ministry of Agriculture, Livestock and Food Supply (MAPA) regularization. More details are provided elsewhere (Tizioto et al., 2013; Cesar et al., 2014).

For the RNA-Seq, a *Longissimus thoracis* (LT) muscle sample of approximately 5 g was collected from the right side of each carcass between the 12th and 13th ribs immediately after the animal’s death and stored in ultra-freezer at −80°C until the analysis. For measurements of intramuscular fat content (IMF, %) and backfat thickness (BFT, mm), a beef sample of the LT muscle (12th–13th ribs, left side of the carcass) was collected 24 h after slaughter. For IMF analysis, beef samples of approximately 100 g were lyophilized and ground, then IMF was achieved using the Ankom XT20 extractor, following the AOCS protocol (AOCS, 2004), a more complete description can be found in Cesar et al. (2014). The BFT was measured by using a graduated ruler, more details in Tizioto et al. (2013).

### High-Density Genotyping Data

The high-density genotyping data acquisition was already described elsewhere (Cesar et al., 2014). Briefly, the genotyping analysis was performed at the Bovine Functional Genomics Laboratory ARS/United States and ESALQ Genomics Center (Piracicaba, São Paulo, Brazil), using the BovineHD 770 k BeadChip (Infinium BeadChip, Illumina, San Diego, CA, United States) following Illumina’s protocol. As a quality control step, SNPs with call rate ≤ 95%, minor allele frequency (MAF) ≤ 5%, located in sexual chromosomes, and those not mapped in the *Bos taurus* ARS-UCD1.2 reference genome were excluded from further analysis.

### RNA-Sequencing

For total RNA extraction, a sample of 100 mg of the LT muscle was processed using the Trizol reagent (Life Technologies, Carlsbad, CA, United States), following the manufacturer’s guidelines. After extraction, RNA integrity was verified using the Bioanalyzer 2100 (Agilent, Santa Clara, CA, United States), and the samples presenting RNA integrity numbers (RIN) greater than 7 were considered for the next analyses. A total of 2 µg of RNA from each sample was used for the cDNA library preparation, according to the protocol described in the TruSeq RNA Sample Preparation kit v2 guide (Illumina, San Diego, CA, United States). The libraries were sequenced using the Illumina HiSeq2500 ultra-high-throughput sequencing system with the TruSeq SBS kit v3-HS (200 cycles), as described in Cesar et al. (2015). All sequencing analyzes were performed at ESALQ Genomics Center (Piracicaba, São Paulo, Brazil). After sequencing, the SeqyClean package v. 1.4.13 (Zhbannikov et al., 2017) was utilized to remove low-complexity reads and the adapters sequences from the library preparation step. For the quality control visualization, FastQC software v. 0.10.1 (https://www.bioinformatics.babraham.ac.uk/projects/fastqc/) was used.

The read alignment against the bovine reference genome *Bos taurus* ARS-UCD1.2 was carried out using STAR (Spliced Transcripts Alignment to a Reference) (Dobin and Gingeras, 2015) v. 2.7 with Ensembl (release 96) gene annotation file. To count the reads, we applied the HTSeq software (Anders et al., 2015) v. 0.11.1 inside STAR. Only reads that were exclusively mapped to known chromosomes were used in this study.

Read counts for each gene were normalized to CPM (Counts per million) using the Bioconductor package edgeR (Robinson et al., 2010), and then the CPM values were log2 transformed (log2-CPM). Genes expressed at a low level or not expressed (log2-CPM value < 0) and expressed in <50% of the samples, were filtered out from the analysis. Additionally, concerning technical biases affecting gene expression, a batch effect correction was performed using the NOISeq R Package (Tarazona et al., 2015) v. 2.16.0. For that, a Principal Component Analysis (PCA) allowed us to explore the dataset and detect possible batch effects. Then, using the ARSyNseq function, we filtered out the noise associated with the batch effect, a combination of flow cell and lane. The datasets analyzed in this study can be found in the European Nucleotide Archive (ENA) repository (EMBL-EBI) under the accession codes: PRJEB13188, PRJEB10898, and PRJEB19421.

### Variant Calling Analysis and SNP Annotation

For the variant calling analysis in the muscle transcriptome, the Genome Analysis Toolkit (GATK) v. 4.1.0.0 was used in the Genomic Variant Call Format (GVCF) mode (Brouard et al., 2019). Using this approach, all genotypes’ types were reported in a final VCF file. The variants were called following the GATK Best Practices, and the Ensembl *Bos taurus* dbSNP (release 96) was used as known variants. The *HaplotypeCaller* algorithm was used to call the variants individually, generating GVCF files for each sample. These files were then merged using the *CombineGVCF* tool, and the joint genotyping analysis was performed using the *GenotypeGVCF*. In the end, a VCF file with all samples genotyped was achieved. After the variant calling, we filtered the SNP for variant quality score (QUAL) ≥ 30 and total depth of coverage (DP) > 10, using BCFtools (Li, 2011) v. 1.9. Moreover, the SNP with call rate < 95%, MAF < 5%, located in sexual chromosomes, and non-biallelic, were removed from the SNP dataset. The variants’ annotation and functional consequences were predicted using the Ensembl Variant Effect Predictor (VEP) (McLaren et al., 2016) v. 95.2.

### Genome-Wide Association Study

Previously to the association study, the filtered SNPs from the RNA-Seq variant calling and the Bovine HD BeadChip were combined into one complete dataset. BEDTools (Quinlan and Hall, 2010) v. 2.27.1 was used to check for common variants (located in the same genome position) in the two datasets. Then, the transcribed variants located in the same spot as those from the Bovine HD BeadChip were removed from the analysis. Thus, a complete dataset containing all SNPs (unique variants) was used for the following analysis.

The GWAS was performed using the GenSel software (Garrick and Fernando, 2013) with a Bayesian approach. First, a Bayes C model was used to estimate the prior genetic and residual variances for each trait with a calculated π (0.9997). Then, these values were used as priors to run a Bayes B model, as previously described in (Cesar et al., 2014; Moreira et al., 2018). The mathematical model was

$$y=\mathrm{Xb}+\;\overset{k}{\underset{j=1}{\sum }}a_{j}\beta_{j}\delta_{j}+e,$$

where ***y*** was the vector of phenotypic values, ***X*** represented the incidence matrix for fixed effects, ***b*** was the vector of fixed effects, *K* was the number of SNP variants (534,928), ***a***_***j***_ was the column vector representing the SNP covariate at locus *j*, assumed to be normally distributed *N* (0, *σ* ^*2*^_*β*_) when *δ*_*j*_ = 1, but *β*_*j*_ = 0 when *δ*_*j*_ = 0, with *δ*_*j*_ being a random variable 0/1, indicating the absence (probability *π*) or presence (probability 1-*π*) of *locus j* in the model, and **e** represented the vector of residuals associated with the analysis. In the model, the contemporary group (animals from the same farm, year, and slaughter date) was set as a fixed effect and hot carcass weight as a covariate.

The GenSel program uses Markov-Chain Monte Carlo (MCMC) to estimate the effect of each SNP among all SNPs in each interaction. In this procedure, 41,000 interactions, with the first 1,000 interactions being discarded, were accumulated to obtain the posterior mean effect for each SNP. A map file was used to position the SNPs into 2,502 non-overlapping 1 Mb SNP windows. Manhattan plots containing the variance explained by each SNP window along the autosomal chromosomes were constructed for each trait. Based on an infinitesimal model (Onteru et al., 2013; Van Goor et al., 2016), it is expected that each window explains 0.04% (100%/2,502) of the genetic variance, thus, windows explaining five times more than the expected (0.20%) were considered as relevant QTL regions.

The Cattle QTL database (Hu et al., 2019) (Cattle QTLdb, release 41) was used to search for known QTL that could be overlapping our relevant QTL regions. For that, we used an in-house R script and the BED file with QTL coordinates according to the *Bos taurus* ARS-UCD1.2 genome, available on Cattle QTLdb. We also checked for previously detected QTL reported by our research group for the interest traits (Tizioto et al., 2013; Cesar et al., 2014). Before this, the LiftOver tool from UCSC Genome Browser (Casper et al., 2018) was used to convert the genome coordinates from the *Bos taurus* UMD3.1 for the current version ARS-UCD1.2. Finally, the genes within the relevant QTL were annotated using the Ensembl Biomart (Ensembl Genes 100).

### Expression Quantitative Trait Loci Identification and Functional Annotation

The R package Matrix eQTL (Shabalin, 2012) v. 2.3 was used to perform cis and trans-eQTL identification, using the complete dataset of SNP and genes with expression values in log2-CPM. The contemporary group was considered in the model for confounding effect correction. According to previous work from our group (Mudadu et al., 2016), no evidence of population stratification was verified in this Nellore population. In the present study, cis-eQTL were defined as SNP located no more than 1 Mb upstream or downstream from the regulated gene, and trans-eQTL as the SNP located more than 1 Mb from the regulated gene. Matrix eQTL tests for associations between SNP genotypes and gene expression using linear regression to associate each gene-SNP pair, considering additive genotype effects. The program also calculates the false discovery rate (FDR), based on Benjamini–Hochberg methodology (Benjamini and Hochberg, 1995), separately for cis and trans-eQTL (Shabalin, 2012). The lists of cis and trans-eQTL (FDR < 0.05) were annotated separately, by using VEP (McLaren et al., 2016) v. 95.2. At last, to verify if the eQTL can be affecting transcription factor (TF), we compared our results with the manually curated list of bovine TFs published by our research group (de Souza et al., 2018).

### Overlap Between Relevant Quantitative Trait Loci and the Expression Quantitative Trait Loci

To verify if the SNPs within relevant QTL regions were also affecting the gene expression, an overlap analysis using those SNPs and the list of cis and trans-eQTL (FDR < 0.05) were performed utilizing the GNU/LINUX environment. Considering all the eQTL (cis and trans) within QTL regions, we used PLINK (Purcell et al., 2007) v. 1.9 to perform linkage disequilibrium (LD) pruning. The parameters applied to variant pruning were pairwise linkage with a minimum *r* ^*2*^ of 0.5 and window size of 100 SNPs, shifting 10 SNPs at each step. Then, carrying out the most representative eQTL (tag-SNP) and the genes regulated by them, we used Cytoscape software (Shannon et al., 2003) to build SNP-gene regulation networks for each trait. Finally, to find the molecular pathways in which the genes regulated and containing the representative eQTL were involved, we used MetaCore software (https://portal.genego.com/) from Clarivate (London, GBR) with the *Mus musculus* database.

## Results

### Phenotypes, High-Density SNP Data, and RNA-Seq Data

In the current study, the Nellore steer population presented mean phenotypic values of 2.93% for IMF and 6.86 mm for BFT, with genomic heritability of 0.25 and 0.18 for IMF and BFT, respectively. From the high-density genotyping SNP data, we obtained a total of 414,879 SNPs that passed all the quality control filters (MAF ≥ 5%; call rate ≥ 95%; not in sex chromosomes; and mapped to the reference genome). Concerning the RNA-Seq data, an average of 18.45 million reads per animal were used as input for mapping to the *Bos tauru*s ARS-UCD1.2 genome. In this analysis, 85.71% of the reads were uniquely mapped to the reference genome. Supplementary Table S1 shows all the mapping reads statistics. Read counts were normalized to counts per million (CPM) and log2 transformed. Filtering steps were applied to remove low-level or not expressed genes and a batch effect correction was performed to minimize technical biases affecting gene expression, totaling 12,991 genes with log2-CPM expression values used in the eQTL identification.

### RNA-Seq Variant Calling Analysis Revealed 120,049 Unique Variants.

The GATK variant calling analysis allowed us to identify 123,300 SNPs that passed all the quality control filters (MAF >5%; call rate > 95%; not in sex chromosomes; and biallelic). There were 3,251 SNPs that overlapped the SNP panel used for genotype (Illumina HD BeadChip). These were used to validate our SNP discovery and revealed 95.85% concordance between genotypes. Variants in the same position as the high-density SNP panel were removed for the next analysis due to their high genotype similarity, and we proceeded with 120,049 unique variants.

Functional annotation analysis of the 120,049 SNPs revealed 9,018 novel variants. Supplementary Figure S1 shows the variant distribution across the 29 *Bos taurus* autosomal chromosomes (BTA) and the most severe consequences predicted by VEP. Most SNPs were in 3′UTR regions (23.24%), followed by downstream genes (18.82%) and intron variants (12.67%). Besides these, 25.28% were synonymous variants, while 9.81% were classified as missense mutations. The SIFT score predicted 2.85% of deleterious SNPs. A complete overview of the results obtained in the annotation analysis can be seen in Supplementary Table S2.

### Twenty-One Relevant Quantitative Trait Loci Associated With Intramuscular Fat and Backfat Thickness

**FIGURE 1:**
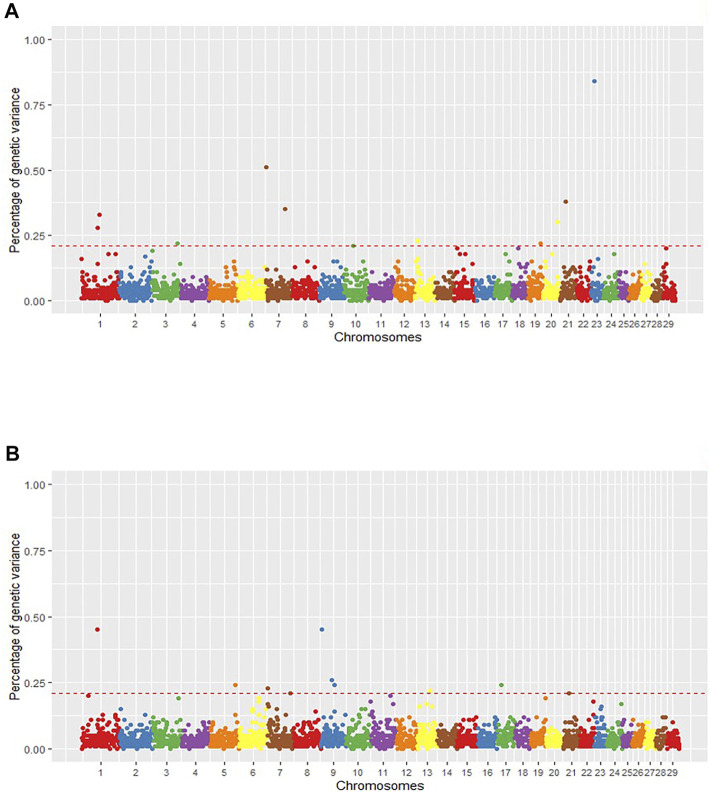
Manhattan plot of the posterior means of the percentage of genetic variance explained by each 1 Mb SNP window across the 29 autosomal chromosomes for intramuscular fat content (IMF) **(A)** and backfat thickness (BFT) **(B)**. The X-axis represents the chromosomes, and the Y-axis, the percentage of genetic variance explained by each SNP window. Red dashed lines delimit the relevant QTL regions.

The Illumina bovine HD SNP panels were designed to cover the entire genomes with equally spaced polymorphic SNPs across different breeds. In the current study, 414,879 SNPs from the *Bos taurus* genome passed all the quality control filters. To empower the SNP database with coding and regulatory variants, we combined the Illumina panel with transcribed variants obtained from the RNA-Seq calling analysis (120,049 SNPs). As a result, for the GWAS, a total of 534,928 SNPs were associated with the phenotypes. Figure 1 shows the Manhattan plots of the proportion of genetic variance explained by the 1 Mb SNP windows for each trait (a complete overview of the SNP windows can be seen in Supplementary Table S3).

Starting with IMF, we found eleven relevant QTL windows positioned on BTA1, 3, 7, 10, 13, 19, 20, 21, and 23 (Table 1). The QTL that explained the highest proportion of the genetic variance of the phenotype (Vg) for IMF (0.84%) was located on BTA23 at 15 Mb. Supplementary Table S4 presents the individual SNP effects within each of the relevant windows. Additionally, using the Ensembl Genes database, we annotated the 155 genes within the relevant QTL associated with IMF (Supplementary Table S5). Then, we consulted the Cattle QTLdb to identify relevant QTL overlapping with known cattle QTL. Our relevant QTL regions were previously associated with carcass and meat quality traits, such as marbling score, fatty acid composition, shear force, and body weight gain in taurine and zebuine breeds (Supplementary Table S6).

**TABLE 1:** Characterization of the relevant QTL regions associated with intramuscular fat content (IMF) and backfat thickness (BFT) in a Nellore cattle population.

Traits	Chr_Mb	First—last position	Proportion Vg (%)	N SNP/window	N Genes/window
IMF	23_15	15000692–15992208	0.84	185	27
	7_2	2003622–2999865	0.51	941	26
	21_22	22001027–22990406	0.38	223	18
	7_81	81002985–81999643	0.35	284	7
	1_75	75000671–75968766	0.33	257	3
	20_62	62002061–62998209	0.30	303	11
	1_67	67000099–67991943	0.28	251	12
	13_13	13000910–13992863	0.23	208	2
	19_54	54022589–54994606	0.22	309	12
	3_108	108002916–108996760	0.22	258	16
	10_37	37022308–37997882	0.21	427	21
BFT	9_4	4040113–4999917	0.45	196	2
	1_63	63008675–63998464	0.45	201	4
	9_46	46000672–46993133	0.26	184	2
	5_104	104002715–104990551	0.24	302	8
	17_18	18002778–18997485	0.24	335	13
	9_55	55005021–55998978	0.24	144	3
	7_3	3000052–3998084	0.23	351	24
	13_52	52001168–52998893	0.22	212	32
	7_96	96002065–96995101	0.21	523	9
	21_23	23014366–23998400	0.21	224	9

Chr_Mb = map position (chromosome and position in Mb) based on the Bos taurus ARS-UCD1.2; Proportion Vg (%) = Proportion of genetic variance explained by 1 Mb SNP, window; N SNP/ window = number of SNP within the genomic region; N Genes/ window = number of genes annotated within the (Ensembl Genes 100) SNP window.

For BFT, we found 10 relevant QTL (Table 1) with 106 genes annotated on these regions (Supplementary Table S5). The QTL that explained the highest proportion of Vg were located on BTA9 at 4 Mb and BTA1 at 63 Mb (0.45% of Vg each). Supplementary Table S7 reports the individual SNP effects within the relevant QTL associated with BFT. Comparing our SNP windows with known bovine QTL (QTLdb) revealed genomic regions associated with body composition and carcass quality traits, like body weight, subcutaneous fat, and shear force in taurine and zebuine cattle breeds (Supplementary Table S6). Moreover, looking for our group’s previously identified QTL, we found two IMF QTL (BTA1_75 and BTA20_62) and three BFT QTL (BTA1_63, BTA7_3, and BTA7_96) encompassing genomic regions associated before with ribeye area, BFT, and fatty acids content (Eicosadienoic acid and Docosahexaenoic acid) in this Nellore population (Tizioto et al., 2013; Cesar et al., 2014).

### Most Cis and Trans-Expression Quantitative Trait Loci Were in Transcribed Regions

**FIGURE 2:**
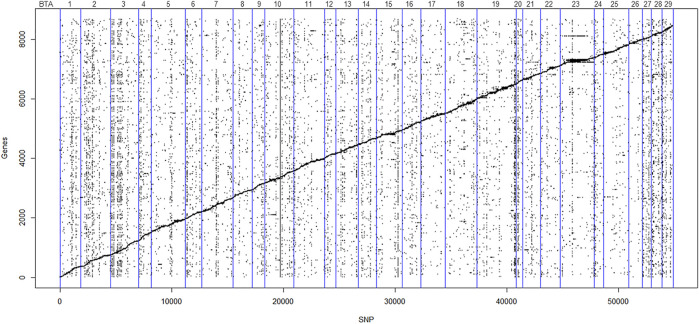
Scatter plot of the affected genes and eQTL (FDR < 0.05). The *Y*-axis represents gene order in relation to chromosome position in the *Bos taurus* genome, and *X*-axis represents the SNP order in relation to chromosome position in the *Bos taurus* genome. Points scattered diagonally indicates cis-eQTL. Points scattered vertically indicate trans-eQTL. The vertical blue lines denote individual autosomal chromosomes.

We identified 71,033 cis-eQTL and 36,497 trans-eQTL (FDR < 0.05) distributed along the genome, with 5,718 SNPs acting both as cis and trans-eQTL. Figure 2 illustrates the eQTL distribution and gene positions (Mb) along the 29 BTAs. Regarding gene regulation, 4,871 genes had their expression affected by cis-eQTL, and among them, 128 were in the curate list of bovine TFs (de Souza et al., 2018). Moreover, 6,370 genes were affected by trans-eQTL, and within them, 259 were TFs. From the total of genes, 2,560 were affected by both cis and trans-eQTL. Supplementary Table S8 displays the complete list of cis and trans-eQTL (FDR < 0.05) and the genes regulated by them, highlighting the TFs.

VEP analysis showed that most of the cis and trans-eQTL were located on BTA19 and BTA23, respectively, while BTA20 had fewer local and distant variants (Supplementary Figure S2). Moreover, most eQTL were variants called from the RNA-Seq data (approximately 92% of the cis and 70% of the trans-eQTL), so the functional annotation results are similar to those presented for the complete dataset of transcript SNPs. Both cis and trans-eQTL were predominantly located in 3’UTR, intronic, and downstream gene regions. Among them, 23.86% and 22.25% were predicted to be synonymous variants, whereas 9.62% and 8.70% were classified as missense for the cis and trans-eQTL, respectively (Supplementary Figure S3).

### Regulatory Polymorphisms Associated With Intramuscular Fat and Backfat Thickness

**FIGURE 3:**
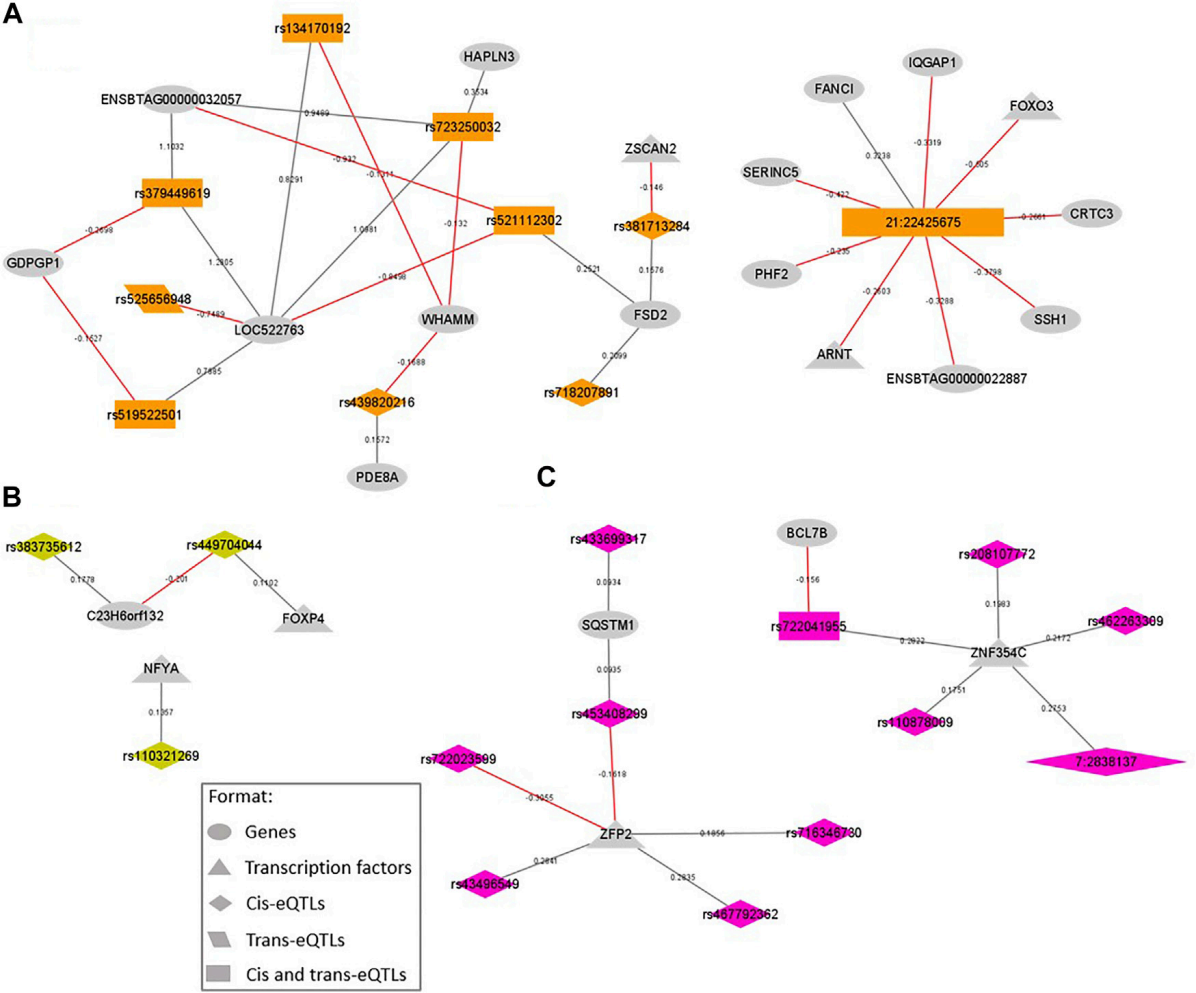
SNP-gene regulation networks representing the eQTL variants located within QTL windows associated with intramuscular fat content (IMF) and the genes regulated by them, focusing on the variants regulating transcription factors (TFs) and their direct connections. The colors are coded by QTL window: **(A)** BTA21_22, eQTL represented in orange; **(B)** BTA23_15, eQTL represented in lightgreen; **(C)** BTA7_2, eQTL represented in pink. All the formats are described in the legend. New variants are represented by chromosome: position. Gray lines represent a positive beta-value and red ones represent a negative beta-value.

To identify eQTL that could be associated with our phenotypes, we overlapped the eQTL and GWAS results. Our analysis revealed that 231 and 109 eQTL variants were located on relevant QTL associated with IMF and BFT, respectively. Within the 231 eQTL associated with IMF (relevant QTL windows described in Table 1), 156 were cis, 26 trans, and 49 cis and trans variants. These regulatory polymorphisms affected the expression of 117 genes, including seven TFs: *ARNT, FOXO3, FOXP4, NFYA, ZFP2, ZNF354C*, and *ZSCAN2*. Besides that, 12 eQTL were missense mutations (Supplementary Table S2), spanning the QTL regions located on BTA1, 3, 7, 10, 19, and 21. Among them, the cis-eQTL rs381713284 (BTA21 at 22 Mb, SIFT = 0.05), the trans-eQTL rs379524684 (BTA3 at 108 Mb, SIFT = 0.03), and the trans-eQTL rs110129172 (BTA10 at 37 Mb, SIFT = 0.02) were deleterious SNP. The SNP rs382320484 (BTA10 at 37 Mb), upstream of the *TMEM87A* and *GANC* genes, affected the expression of the largest number of genes (12 genes in trans and two genes in cis); followed by the novel SNP 21:22425675 (chromosome: position), a synonymous variant located on the *ZNF592* gene, that affected the expression of nine genes (seven genes in trans and two genes in cis), being two TFs (*FOXO3* and *ARNT*). Moreover, within the genes regulated by eQTL associated with IMF, the two genes regulated by the larger number of eQTL were the pseudogene *ENSBTAG00000052719* (BTA7) by 21 cis-eQTL, and the *TMEM87A* (BTA10) by 13 cis-eQTL. In Figure 3, we presented the SNP-gene regulation networks for the eQTL associated with IMF, focusing on the eQTL affecting TFs and their direct connections.

**FIGURE 4:**
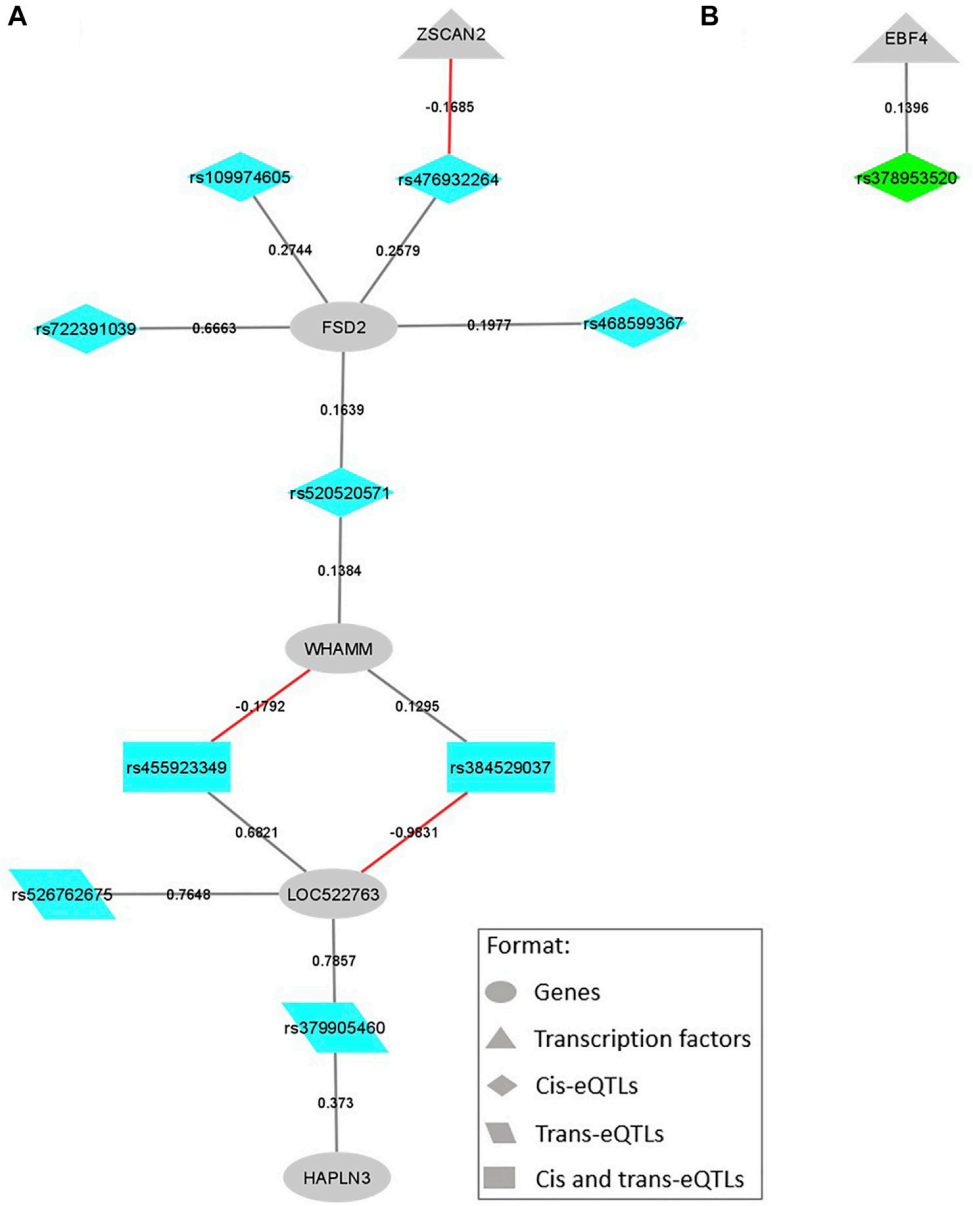
SNP-gene regulation networks representing the eQTL variants located within QTL windows associated with backfat thickness (BFT) and the genes regulated by them, focusing on the variants regulating transcription factors (TFs) and their direct connections. The colors are coded by QTL window: **(A)** BTA21_23, eQTL represented in blue; **(B)** BTA13_52, eQTL represented in green. All the formats are described in the legend. Gray lines represent a positive beta-value and red ones represent a negative beta-value.

Associated with BFT, there were 74 cis, 20 trans, and 15 both cis and trans-eQTL. These variants were located on the SNP windows BTA9_46, BTA5_104, BTA17_18, BTA7_3, BTA13_52, BTA7_96, and BTA21_23 (see Table 1). Together, the 109 eQTL affected the expression levels of 54 genes, among them two TFs, the *EBF4* and *ZSCAN2*. The genes regulated by the larger number of eQTL were *NDUFC1* (BTA17), which is affected by nine eQTL (cis and trans), and the novel gene *ENSBTAG00000025383* (BTA12), which is affected by eight eQTL (cis and trans). Figure 4 illustrates the SNP-gene regulation networks for the eQTL associated with BFT, focusing on the eQTL affecting TFs and their direct connections. As occurred for IMF, we found 12 missense variants within our relevant eQTL. These variants were located on BTA5, 7, 13, 17, and 21 and all of them were classified as tolerated by VEP (without deleterious effects). The complete list of the eQTL spanning IMF and BFT relevant QTL, their regulated genes, and beta-values (effect size and direction) are presented in Supplementary Table S9.

**FIGURE 5:**
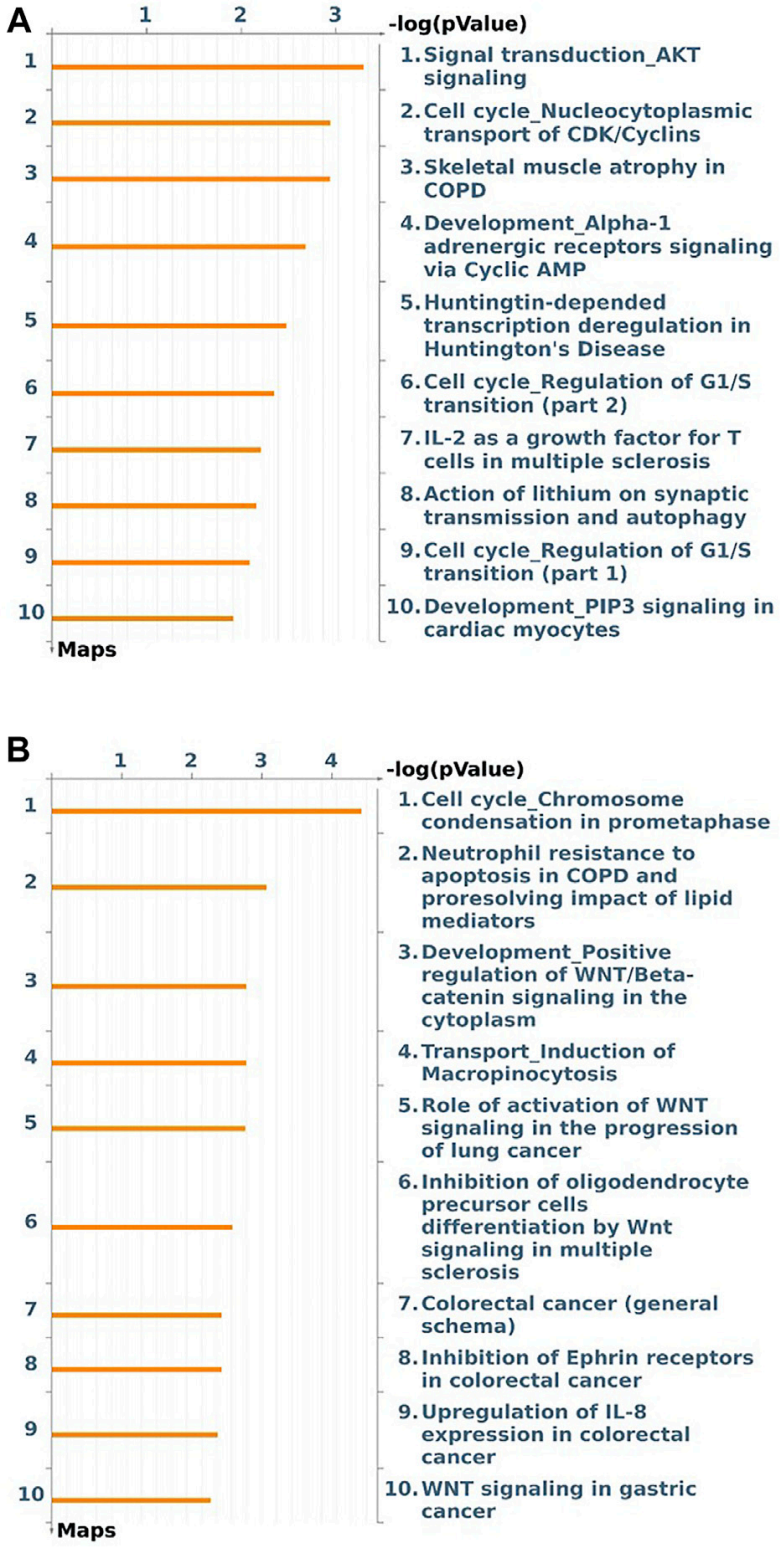
Top 10 pathway maps enriched for the genes regulated and containing the eQTL encompassing relevant QTL regions associated with intramuscular fat content (IMF) **(A)** and backfat thickness (BFT) **(B)** in a Nellore cattle population.

At last, to investigate if the genes regulated and containing eQTL associated with the interest traits were involved in lipid metabolism-related pathways, we did an enrichment analysis of these genes (135 and 74 genes for IMF and BFT, respectively). This analysis revealed some interesting pathways, such as signal transduction, cell cycle, development, and transport, underlining the AKT and WNT signaling. Figure 5 shows the top ten Pathway Maps [−log (p-value)] enriched for the genes related to IMF and BFT.

## Discussion

In the present study, we used an SNP dataset constituted by RNA-Seq variants and a high-density genotyping panel to perform an integrative analysis between GWAS and eQTL. The idea was to find gene expression regulatory polymorphisms associated with intramuscular fat and backfat thickness in bovine. Backfat thickness and intramuscular fat deposition are of economic importance to the beef cattle industry. The BFT is the best predictor of overall fatness in the animal’s body, impacting carcass cutability and meat yield (Yokoo et al., 2008; Lopes et al., 2012). The IMF is positively correlated with beef tenderness, a meat quality trait that strongly affects consumer satisfaction and repurchase decision (Park et al., 2018). Moreover, the beef fatty acids composition is associated with human health (Troy et al., 2016). Herein, the mean phenotypic values of IMF and BFT were higher than those presented in the literature for Nellore cattle, with mean values ranging from 2 to 5 mm for BFT, and around 1% for IMF (Yokoo et al., 2010; Borges et al., 2014; Fernandes Júnior et al., 2016). Nevertheless, selection for these traits can be difficult as they are expressed later in the animal’s life. In this way, identifying genes and genetic markers with causative effects over these traits will help improve breeding progress in bovine (Fernandes Júnior et al., 2016).

GWAS has been widely used to identify genetic variants and putative candidate genes associated with complex phenotypes (Cesar et al., 2014; Fernandes Júnior et al., 2016; Raza et al., 2020; Martins et al., 2021). However, panels used for GWAS studies are not designed to have causative SNP since the goal is to have informative markers across the genome (Tam et al., 2019). In the current study, we used RNA-Seq-based SNP to empower the high-density genotyping panel. According to Suárez-Vega et al. (2015), calling variants from RNA-Seq raises the chances of discovering causative mutations harboring or neighboring QTL and can provide a better understanding of the regulatory mechanisms underlying eQTL.

With the incorporation of RNA-Seq-based SNP, we were able to identify QTL associated with IMF and BFT that better explained the genetic variance of the phenotype compared with previous works (Tizioto et al., 2013; Cesar et al., 2014), even using a minor subset of the population (193 animals that have RNA-Seq information). Tizioto et al. (2013), using only the high-density SNP panel (Bovine HD 770 k) and 536 animals from the same population, found that the highest effect QTL for BFT was located on BTA11 and only explained 0.36% of Vg, with genomic heritabily (h^2^) of 0.21. Here, the two QTL with the highest effect for BFT were detected on BTA9 and BTA1, and each explained 0.45% of Vg, with a slightly smaller h^2^ of 0.18. In another study using the same population (Cesar et al., 2014) with 386 Nellore steers with phenotypes for IMF and the HD 770 k chip, the highest effect QTL for IMF (located on BTA10) explained 0.66% of Vg (h^2^ = 0.25), while in this study, the highest effect QTL was detected on BTA23 and it explained 0.84% of Vg (h^2^ = 0.25).

Complementary to the GWAS analysis, we identified 71,033 cis-eQTL and 36,497 trans-eQTL in this Nellore population, most of them from the RNA-Seq dataset, showing the importance of adding transcribed variant calling information on eQTL mapping, as stated by Suárez-Vega et al. (2015). Most of the SNPs were in 3′UTR, introns, and downstream gene regions, but some were missense variants (about 9%). It is important to keep in mind that the polymorphisms identified in this study may not be the causative mutation but could be in linkage disequilibrium with the causative one (Mueller, 2004), thus explaining how a missense SNP could be associated with gene expression variation.

There are several advantages in integrating GWAS and eQTL data. Expression quantitative trait loci are essential for understanding the genetic basis of cellular processes and complex traits (Lee, 2018). Moreover, eQTL are essential for the functional interpretation of trait-associated polymorphisms and identification of genes with expression levels associated with complex phenotypes (Westra and Franke, 2014; Littlejohn et al., 2016; Cai et al., 2019). In the current work, we focused on the overlap between the eQTL and relevant QTL regions for each trait, concentrating on the mutations affecting the expression of transcription factors.

### Intramuscular Fat Content

Seven TFs were regulated by eQTL harbored on QTL regions associated with IMF. Transcription factors are cellular components that exert an essential role in regulating gene expression (Vaquerizas et al., 2009). Studying how these components are regulated is attractive to a more in-depth investigation of gene expression patterns during development and terminally differentiated cells (Calkhoven and Ab, 1996). Thus, we first highlight here the *FOXP4* TF, positively regulated by a cis-eQTL (rs449704044) located downstream of the novel gene *ENSBTAG00000054479*, and harbored on the most relevant QTL for IMF (BTA23 at 15 Mb), that explained 0.84% of Vg. The Forkhead box (Fox) family genes are expressed in various tissues, acting both in developmental processes as in tissue maintenance during adult life (Golson and Kaestner, 2016; Zhu, 2016). The same cis-eQTL (rs449704044) negatively affected the expression levels of the *C23H6orf132* gene (Figure 3B). Although there is a lack of information about the *C23H6orf132* gene function in cattle, according to the GeneCards human database (Stelzer et al., 2016), Fox TFs were predicted to bind TFBS (transcription factor-binding sites) of this gene. The *C23H6orf132* was also regulated by the rs383735612 cis-eQTL, located in the 3’UTR region of the *TAF8* gene. The *TAF8* is an important modulator of early adipogenesis. Because of its histone fold domain, *TAF8* can inhibit adipogenesis by specifically downregulating the expression of the peroxisome proliferator-activated receptor γ (PPARγ) and the CCAAT enhancer-binding protein α (C/EBPα), major promoters of adipogenesis (Guermah et al., 2003; Lefterova et al., 2014). Wang et al. (2013) found *TAF8* as a target gene of a miRNA highly expressed in subcutaneous fat of beef cattle, and further, enriched for the “regulation of fat cell differentiation” biological process, corroborating its relevance in controlling fat deposition in bovine. Another TF affected by an eQTL located on BTA23 was *NFYA* (see Figure 3B), also known as CCAAT-box Binding Protein A (CBP-A). This TF plays a role in the early development of adipocytes, as well as, is essential for leptin gene expression (Lu et al., 2015). The knockout of this gene in mice resulted in lipodystrophy with a progressive loss of adipose tissue (Lu et al., 2015).

We also emphasize another member of the Forkhead box family, the *FOXO3.* This TF is negatively regulated in trans by a synonymous novel SNP (21:22425675) located on the exonic region of *ZNF592* gene. This SNP also regulates six more genes in trans and two genes in cis (Figure 3A). In the skeletal muscle, FoxO genes, including *FOXO3*, are responsible for switching carbohydrate to lipid as an energy source during starvation periods and can interact with the PPARγ (Gross et al., 2008). In previous work, Cesar et al. (2016), identified *FOXO1* and *FOXO3* as upstream regulators of gene expression in the skeletal muscle of Nellore cattle influenced by a variation in oleic acid content. Gui and Jia (2018) found a polymorphism in the 3’UTR region of *FOXO1* associated with IMF in Qinchuan cattle. The authors hypothesized that the variant could affect *FOXO1* expression levels through miRNA activity, thus modulating changes in fatty acid metabolism. This hypothesis corroborates our findings of a regulatory SNP affecting the expression levels of a FoxO gene associated with IMF in beef cattle.

From the list of nine genes regulated by the aforementioned novel SNP 21:22425675 (Figure 3A), there is one more TF, the *ARNT*, and some noteworthy genes, such as the *CRTC3, IQGAP1,* and *SSH1*. The *ARNT*, negatively regulated in trans, is a nuclear translocator that binds the aryl-hydrocarbon receptor (AhR). This binding forms a heterodimer that attaches to Xenobiotic/Dioxin response element sequences (XRE/DRE) of different target genes, activating mRNA transcription (Ishihara et al., 2018). Conversely, the *CRTC3* and the *IQGAP1* were regulated in cis by this eQTL. The first one, *CRTC3* is part of the cAMP responsive element-binding protein (CREB)-regulated transcription coactivator (CRTC) family and plays an important role in lipid and glucose metabolism (Liu et al., 2020; Liu et al., 2021). Studying IMF deposition, Liu et al. (2020) overexpressed *CRTC3* in porcine IMF adipocytes and observed a faster accumulation of lipid droplets in cells together with an upregulation of important fat metabolism genes, such as perilipin, PPARγ, C/EBPα, leptin, and FABP4 (Fatty acid-binding protein 4). In a more recent work, Liu et al. (2021), demonstrated that the overexpression of *CRTC3* changes the metabolic profile in intramuscular adipocytes, and also promotes adipogenic differentiation of intramuscular and subcutaneous adipocytes through the calcium signaling pathway. We also highlight that in the same genomic region (BTA21, 22 Mb), we found two other eQTL (rs379449619 and rs525656948) located on the *CRTC3* gene associated with IMF in our population, emphasizing its relevance for IMF molecular regulation. As for *IQGAP1,* this gene encodes a ubiquitously expressed scaffolding protein implicated in several cellular processes, including mitogen-activated protein kinase and AKT signaling cascades (Erickson and Anakk, 2018; Hedman et al., 2021). Studies investigating the loss of this protein in mice demonstrated a reduced PPARγ activity, as well as, a defective transcription of gluconeogenesis and fatty acid synthesis genes (Erickson and Anakk, 2018; Hedman et al., 2021). Finally, the *SSH1* gene, known by its function in cytoskeleton organization and cell migration, was previously found associated with body fat in humans (Gomez-Santos et al., 2011). In earlier work from our group, studying the proteomic profile of high and low IMF Nellore cattle, we found SSH1 protein downregulated in the group with higher values of IMF, suggesting its involvement in the cellular rearrangement needed for adipocyte growth (Poleti et al., 2018).

Still focusing on the genomic window of BTA21, we emphasize the zinc-finger gene *ZSCAN2*, a TF negatively affected in cis by a deleterious SNP (rs381713284). Deleterious mutations can be defined as genetic alterations that raise individual susceptibility or predisposition to diseases/disorders. These variations often occur in coding regions and are typically missense, causing changes in the amino acid sequence, and consequently, in the protein (Plekhanova et al., 2018). According to van Strien (2018), it is coherent that variants causing changes in the protein product (missense) also can affect the expression levels of the gene coding for the protein (cis-effects) or on other genes (trans-effects). Here the rs381713284 (located on the *WDR73* gene), which changes an Arginine for a Cysteine in the protein sequence, was the only missense eQTL presenting cis-effects. Leal-Gutiérrez et al. (2020) found that a higher expression level of *WDR73* is associated with a lower meat quality index (lower marbling score, higher connective tissue content, tougher and dryer meat) in Angus-Brahman steers. Although missense mutations on the *WDR73* gene were already reported in humans associated with neuro disorders (Jiang et al., 2017), as far as we know, in cattle, there were no previous reports of deleterious SNPs on this gene associated with IMF.

Other TF identified herein affected by eQTL associated with IMF (Figure 3C) were the *ZFP2* and the *ZNF354C*, both members of the zinc-finger family. Together, *ZFP2* and *ZNF354C* were affected by 10 cis-eQTL located on BTA7 at 2 Mb, the second most relevant QTL for IMF, explaining 0.51% of Vg. Zinc-finger proteins are a large family of TFs characterized by a zinc-finger domain in their structure. They are ubiquitously expressed in eukaryotic genomes, participating in growth regulation, cell development, immunity, and signal transduction pathways. During adipogenesis, zinc-finger TFs are key molecules in preadipocytes differentiation and adipocyte determination. Moreover, zinc-finger TFs can both activate and inhibit the PPARγ and C/EBPs (Wei et al., 2013; Cassandri et al., 2017). Most of the eQTL affecting these two zinc-finger gene expression levels were located in novel genes, except for two of them (rs208107772 and rs462263309) located on the exonic region of *ADAMTS2*. The A Disintegrin and Metalloproteinase with Thrombospondin motifs (ADAMTS) family exerts a principal role in the extracellular matrix (ECM) maintenance and remodeling, mainly by participating in collagen biosynthesis (Kelwick et al., 2015). Lee et al. (2010) found the *ADAMTS4* overexpressed in the *Longissimus dorsi* muscle of Korean cattle presenting high IMF. Cao et al. (2017) identified the *ADAMTS2* gene as differentially methylated and differentially expressed when comparing two sheep breeds known by their different carcass weight and meat yield, confirming that this gene may indirectly affect marbling through collagen synthesis.

Finally, the enrichment analysis of the genes regulated and containing eQTL associated with IMF revealed that those genes were involved in signal transduction, cell cycle, and development pathways, like the AKT signaling (Figure 5A). The AKT or PI3K-AKT signaling is an intracellular pathway essential for signal transduction, cell proliferation, apoptosis, and metabolism (Yun et al., 2020). Furthermore, AKT plays a crucial role in adipocyte differentiation. AKT can drive fat production and promote adipogenesis through phosphorylation of substrates, such as Fox family members (Kim et al., 2010; Wang et al., 2020; Yun et al., 2020). Corroborating these findings, in the current study, the *FOXO3* TF, negatively regulated by the 21:22425675 eQTL, was enriched for the Signal transduction AKT signaling. Besides that, although not enriched in this pathway, the *IQGAP1* gene, affected by the same eQTL plays a role in AKT signaling cascades. Li et al. (2017), studying transcriptional differences in pigs in high and low BFT groups, found the PI3K-AKT signaling pathway enriched for differentially expressed liver miRNAs in these animals. Liang et al. (2017), also found the PI3K-AKT pathway related to lipid metabolism and milk fat formation in Holstein cows. These findings indicate that the genes being regulated/containing eQTL associated with IMF participate in relevant lipid-metabolism pathways.

### Backfat Thickness

Regarding the eQTL harbored on relevant QTL associated with BFT, most of them were cis-eQTL. Among the affected genes, *EBF4* and *ZSCAN2* are part of the list of bovine curated TFs (de Souza et al., 2018). The first one, *EBF4*, is positively regulated by a single cis-eQTL (rs378953520) harbored on the *PCED1A* gene (Figure 4B), and located on the QTL region of BTA13 at 52 Mb (0.22% Vg). This gene is a helix-loop-helix TF, member of the early B cell factor (Ebf) gene family, that in vertebrates is composed of four members, *EBF1* to *4*. The *EBF1* and *2* participate in the adipogenesis process by playing critical roles during the transcriptional adipogenic cascade (Akerblad et al., 2002; Jimenez et al., 2007). Recently, Cao et al. (2021), in a genome-wide DNA methylation study associated with body fat traits in healthy adult humans, identified a differential methylated position associated with body mass index in the 3’UTR region of the *EBF4* gene, suggesting that this gene could be a target for future obesity risk research. About the gene harboring this mutation, the *PCED1A* is an esterase part of the GDSL/SGNH superfamily, and is expressed in multiple tissues (Maynard et al., 2018). Despite limited studies about the specific functions of this gene, Maynard et al. (2018) indicated a potential structural function at the cell membrane and/or the ECM. As already mentioned, structural genes may exert a function in the cellular rearrangement during adipocyte expansion.

Concerning *ZSCAN2,* this TF is negatively regulated by a cis-eQTL (rs476932264) in the *FSD2* gene (Figure 4A), located on BTA21 at 23 Mb, a region that explained 0.21% of Vg in GWAS analysis. Interestingly, *ZSCAN2* expression levels were also negatively regulated by a missense cis-eQTL associated with IMF (Figure 3A). This gene belongs to the zinc-finger family of TFs, exerting a role during adipocytes differentiation and determination, and may promote and inhibit PPARγ and C/EBPs expression (Wei et al., 2013; Cassandri et al., 2017). Regarding *FSD2*, this gene not only harbors the rs476932264 cis-eQTL, but also the rs109974605 and rs379905460 (Figure 4A). The rs476932264 and rs109974605 are part of the five cis-eQTL affecting their own gene expression levels. While, rs379905460 is a trans-eQTL affecting the expression of both *LOC522763,* a cattle novel gene, and *HAPLN3,* a hyaluronan and proteoglycan-binding link protein gene involved in integrity maintenance and binding functions of the ECM (Spicer et al., 2003). In previous work from our group, a SNP in the *FSD2* gene was already associated with meat color in this Nellore cattle population (Tizioto et al., 2013). Moreover, Lim et al. (2016), indicated it as a potential determinant of overall meat quality in pigs. The authors tested the association of haplotypes produced by *FSD2* SNP and meat quality traits in Berkshire pigs, showing significant associations of the haplotypes with moisture, crude protein levels, color, and IMF content.

We also highlight here the genes *ENSBTAG00000025383* and *NDUFC1*. These two genes were affected by 8 and 9 eQTL associated with BFT, respectively, being the most regulated ones. Although there is a scarcity of information about the novel gene *ENSBTAG00000025383*, it is part of the *NDUFC1* Gene Tree, according to the Ensembl database. The NADH-ubiquinone oxidoreductase (NDUF) enzymes are components of the Complex I oxidative phosphorylation system in mitochondria. In mammals, almost all the ATP molecules required by the cells are generated by oxidative phosphorylation in the mitochondrial respiratory chain (Papa et al., 2002). Kim et al. (2009) and Karisa et al. (2013) found NDUF genes related to beef cattle growth and fat deposition traits (ribeye area and marbling). Corroborating these findings, Jeong et al. (2013) studied transcriptome alterations on the skeletal muscle of castrated Korean cattle that drives IMF deposition and found upregulated NDUF genes enriched for the oxidative phosphorylation process. Cesar et al. (2016), working with this Nellore population, identified NDUF genes differentially expressed in the skeletal muscle associated with fatty acid content. Even though none of the cited studies found these genes associated with the BFT, we have enough evidence to support them as candidate genes.

Lastly, the enrichment analysis of the genes regulated/containing eQTL encompassing relevant QTL associated with the backfat thickness revealed cell cycle, development, and transport pathways. We highlighted the WNT signaling, an important regulator of adipogenesis (Bowers and Lane, 2008). This pathway regulates mesenchymal stem cells, promotes osteogenesis and myogenesis, and inhibits adipogenesis through deacetylation of PPARγ and C/EBPα promoters, and also, by blocking their expression (Bowers and Lane, 2008). In a previous work (Silva-Vignato et al., 2017), studying the skeletal muscle transcriptome of a subset of this Nellore population with extreme values for BFT (high and low groups), we found the WNT signaling enriched for an upregulated gene in the low BFT group. Similarly, Li et al. (2017), working with a pig population with divergent BFT phenotypes (high and low groups), identified the WNT signaling enriched for the differentially expressed miRNAs.

In conclusion, combining RNA-Seq information (expression and SNP) with a high-density genotyping panel, allowed us to identify relevant genomic regions and regulatory polymorphisms associated with intramuscular fat and backfat thickness of Nellore cattle. Within the genes regulated by eQTL associated with the interest traits, we highlight that the transcription factors *FOXP4, FOXO3, ZSCAN2,* and *EBF4* are involved in lipid metabolism-related pathways and may regulate major adipogenesis genes, such as the PPARγ and C/EBPα. We also reported for the first time, a missense cis-eQTL in the *WDR73* gene associated with the intramuscular fat content. These findings help us to improve our knowledge about the genetic architecture that controls economically important carcass and meat quality fat traits in bovine.

## Data Availability

The datasets presented in this study can be found in online repositories. The names of the repository/repositories and accession number(s) can be found in the article.

## Abbreviations

BFT, backfat thickness; ECM, extracellular matrix; eQTL, expression quantitative trait loci; IMF, intramuscular fat; LT, Longissimus thoracis; QTL, quantitative trait loci; TF, transcription factor.
