# Supplementary material for: Integrative Analysis Between Genome-Wide Association Study and Expression Quantitative Trait Loci Reveals Bovine Muscle Gene Expression Regulatory Polymorphisms Associated With Intramuscular Fat and Backfat Thickness
Source: Front Genet. 2022 Aug 4;13:935238. doi: 10.3389/fgene.2022.935238 (PMC9386181; doi:10.3389/fgene.2022.935238)
Supplement: Supplementary file 5 [file DataSheet1.PDF]

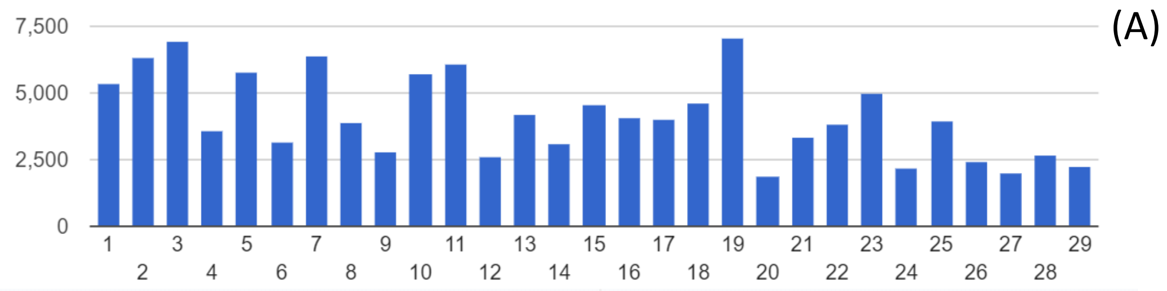

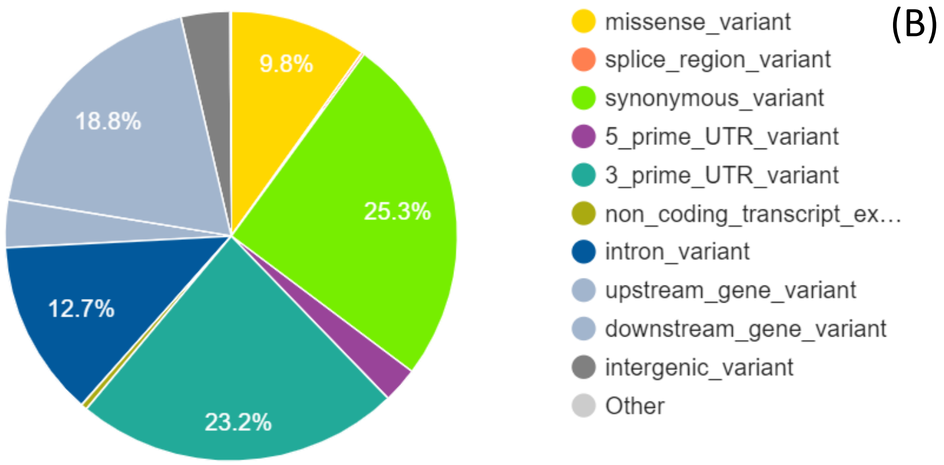

Supplementary Figure 1. Chromosome distribution (A) and most severe consequences (B) of the transcribed single nucleotide polymorphisms (SNPs) predicted by the Variant Effect Predictor in the *Longissimus thoracis* muscle of a Nellore cattle population. In part A, the X-axis represents the chromosomes, and the Y-axis the number of variants per chromosome.

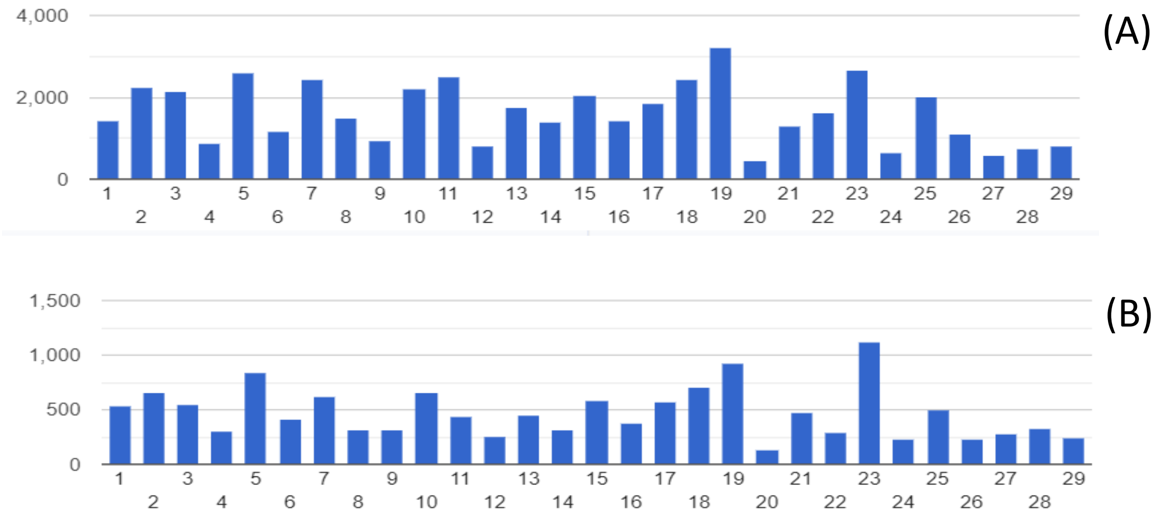

Supplementary Figure 2. Distribution of cis (A) and trans-eQTLs (B) across the 29 *Bos taurus* autosome chromosomes in the *Longissimus thoracis* muscle of a Nellore cattle population. The X-axis represents the chromosomes, and the Y-axis the number of variants per chromosome.

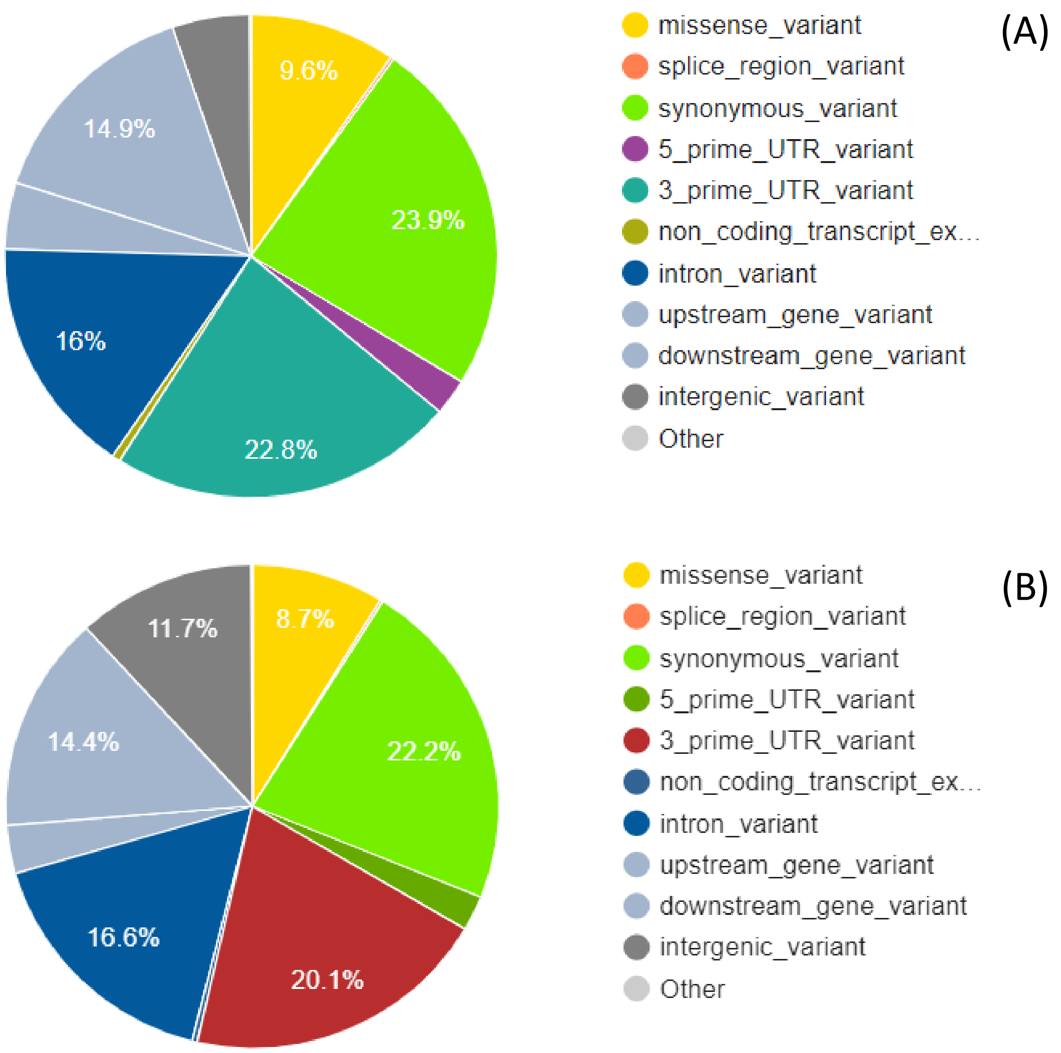

Supplementary Figure 3. Most severe variant consequences predicted by the Ensembl Variant Effect Predictor for cis (A) and trans-eQTLs (B) identified in the *Longissimus thoracis* muscle of a Nellore cattle population.
